# Howard Hospital

**Published:** 1860-09

**Authors:** 


HOWARD HOSPITAL.—Dr. S. W. Gross has been appointed one of the Surgeons to this institution, to take the Department of Diseases of the Genito-Urinary Organs, and Dr. Charles Neff has been appointed to the Department of General Surgery.

